# Chronic kidney disease among patients from gold-mining communities in southeastern Senegal: A call for further investigation

**DOI:** 10.4102/jphia.v17i1.2047

**Published:** 2026-06-25

**Authors:** Modou Ndongo, Ibrahima L. Sarr, Sidy M. Seck

**Affiliations:** 1 Department of Nephrology and Hemodialysis, Regional Hospital of Kedougou, Kedougou, Senegal; 2 Department of Nephrology and Dialysis, Military Hospital of Ouakam, Dakar, Senegal; 3 Department of Nephrology, Faculty of Health Sciences, Gaston Berger University, Saint-Louis, Senegal

**FIGURE 1:**
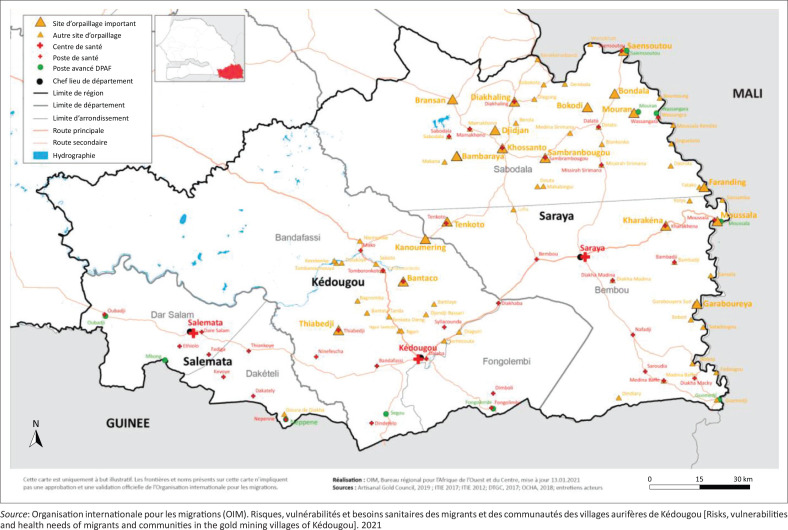
Geographic distribution of gold-mining sites in the Kedougou region.^5^

Dear Editor,

Chronic kidney disease (CKD) is an increasing public health challenge in sub-Saharan Africa, where its determinants remain insufficiently characterised, particularly in rural and environmentally exposed populations. In southeastern Senegal, the Kedougou region has experienced rapid expansion in artisanal and industrial gold mining over the past decade. In May 2023, the region established its first nephrology and haemodialysis unit at the Amath Dansokho Regional Hospital, providing an opportunity to describe the geographic origin of patients initiating chronic dialysis. Gold-mining activities in Kedougou are concentrated in specific municipalities, particularly within the Saraya and Kedougou departments (Figure 1). Major mining areas include Bembou, Sabodala, Khossanto and Tomboronkoto, whereas the Salemata department has comparatively limited mining activity. Between August 2023 and August 2025, according to data from the haemodialysis registry of the nephrology and haemodialysis unit at Amath Dansokho Regional Hospital, 59 patients initiated chronic haemodialysis for end-stage kidney disease (ESKD). Among them, 38 (64.4%) were residents of the Kedougou region (Table 1). Among these regional residents, 19 patients (50.0%) originated from the Kedougou department, 16 (42.1%) from Saraya and 3 (7.9%) from Salemata.

**TABLE 1:** Overrepresentation of dialysis patients in mining and non-mining communes in the Kedougou region (*n* = 38).

Commune	Gold-mining activity	Dialysis patients (%)	Population (%)†	Ratio††
Bembou	Yes	26.3	10.9	2.4
Tomboronkoto	Yes	15.8	7.5	2.1
Sabodala	Yes	10.5	7.4	1.4
Kedougou	No	21.1	24.2	0.9
Saraya	No	2.6	1.8	1.4

† , Population data from the National Agency for Statistics and Demography (ANSD), 2023.^6^

†† , Proportion of dialysis patients divided by the proportion of the population.

An apparent geographic concentration of dialysis patients was observed in several mining municipalities. Bembou accounted for 26.3% of dialysis patients in the region, despite representing 10.9% of the regional population, while Tomboronkoto accounted for 15.8% of dialysis patients and 7.5% of the population. Similar observations were noted in Sabodala. In contrast, several municipalities with limited mining activity reported few or no dialysis patients despite larger population shares.

These descriptive observations raise concerns about potential environmental and occupational contributors to CKD in mining communities. Artisanal gold extraction in the region commonly involves the use of mercury and other potentially nephrotoxic substances, which may contaminate water, soil and food chains. Chronic exposure to such toxicants has been associated with kidney damage in other mining contexts.^1,2^ Environmental dispersion of nephrotoxic metals from mining activities may also affect nearby communities through chronic contamination of water and agricultural ecosystems.^3,4^ This raises concern that the renal health impact of artisanal mining may extend beyond occupationally exposed workers to the broader rural population.

However, these findings should be interpreted cautiously because of the descriptive, single-centre design, the limited sample size and the possibility of healthcare-access biases. No causal relationship can be inferred from these observations. Nevertheless, the apparent spatial overlap between mining areas and the origins of dialysis patients highlights the need for further longitudinal investigation to ascertain causation.

Population-based studies integrating environmental monitoring, biomarker assessment and spatial epidemiology are needed to better characterise the burden of CKD and potential mining-related exposures in southeastern Senegal.

In conclusion, the apparent clustering of ESKD cases in gold-mining municipalities of southeastern Senegal raises concern about potential environmental and occupational contributors to CKD. While causal inference is not possible from these descriptive observations, strengthening environmental surveillance, occupational health measures, and early CKD detection in mining communities may represent important public health priorities.
